# Complication after vein of Marshall ethanol ablation: A case of acute cardiogenic shock and death

**DOI:** 10.1016/j.hrcr.2025.05.029

**Published:** 2025-06-06

**Authors:** Ghazaleh Goldar, Aaron Sifuentes, Sean Byrnes, Peter Farjo, Paari Dominic

**Affiliations:** Division of Cardiology, University of Iowa Hospitals and Clinics, Iowa City, Iowa

**Keywords:** Vein of Marshall ethanol ablation, ST-segment elevation, Cardiogenic shock, Atrial fibrillation, Pulmonary vein isolation

**Key Teaching Points**

1. Severe complications and unclear cause of hemodynamic collapse: Although vein of Marshall (VOM) ethanol infusion is generally well tolerated, this case highlights the rare but severe risk of acute cardiogenic shock, with no clear mechanism identified despite extensive testing. Possible mechanisms include coronary thrombus, vasospasm, atrioventricular malformation, or another unidentified pathway.
2. Higher ethanol dose: A higher dose of ethanol was used in this case than in the initial trials, given that high-volume centers have adopted higher doses owing to the VOM’s anatomic characteristics, which allow for more controlled delivery. However, to ensure safety, it is recommended to use the lowest effective dose, administered in carefully divided increments, to minimize risks and ensure patient safety. Understanding the patient’s anatomy through methods such as computed tomography or electroanatomic mapping can potentially help tailor the ethanol volume and infusion rate to reduce risks and enhance safety.
3. Importance of close hemodynamic monitoring and access to emergent interventions: During VOM ethanol infusion, continuous monitoring is crucial, and operators should be prepared for emergent intervention, including mechanical circulatory support devices, to prevent catastrophic outcomes.

## Introduction

Vein of Marshall (VOM) ethanol infusion has emerged as an effective adjunctive therapy for atrial tachyarrhythmias, particularly in patients with persistent atrial fibrillation (AF). Owing to its insulation by surrounding fat, radiofrequency ablation of the VOM bundle has long been challenging. Chemical ablation via retrograde ethanol infusion offers a more efficient approach, improving arrhythmia suppression.^1^ Recent multicenter trial data suggest that VOM ethanol infusion, beyond pulmonary vein isolation, reduces the recurrence of atrial tachyarrhythmias in persistent AF.^2^

Although generally well tolerated, infrequent complications have been reported, including VOM perforation, pericarditis, cardiac tamponade, stroke, anaphylactic shock, high-degree atrioventricular (AV) block, and unintended left atrial appendage isolation.^3^ Serious adverse events have been rare. However, to date, no case of cardiogenic shock after VOM ethanol infusion has been described. Here, we present the first reported case, highlighting the need for awareness of this potential complication.

## Case report

**Figure 1:**
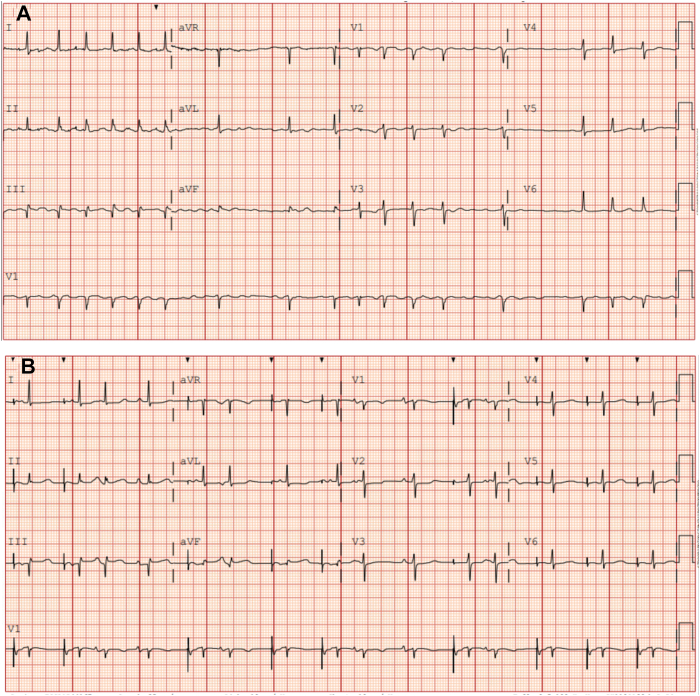
**A:** The baseline electrocardiogram before the procedure. **B:** Electrocardiogram obtained during the vein of Marshall ablation procedure, notable for ST elevations in the inferior leads.

A 78-year-old woman presented for an elective pulmonary vein isolation using pulse field ablation and VOM ethanol ablation. After standard access and preparation, coronary sinus angiography was used to identify the VOM, and 10 mL of 100% ethanol was infused over 3–4 minutes. Before almost the entire dose of ethanol was injected, the patient developed inferior ST elevations (Figure 1) and profound hypotension, requiring vasopressors. Transseptal access was not achieved, and pulse field ablation was not performed owing to clinical deterioration.

### Medical history

The patient had a medical history of heart failure with mildly reduced ejection fraction (EF) with an EF of 45%–50%, mildly reduced right ventricular (RV) function, persistent nonvalvular AF, and hyperlipidemia. She did not have any known history of coronary artery disease and had a recent negative nuclear stress test.

### Differential diagnosis

Differential diagnoses for ST-segment elevation during AF ablation included air embolism, AV malformation causing alcohol entry into the arterial system, acute coronary thrombus, or coronary vasospasm, the latter of which has been reported in only 1 other case.^4^

### Investigations

**Figure 2:**
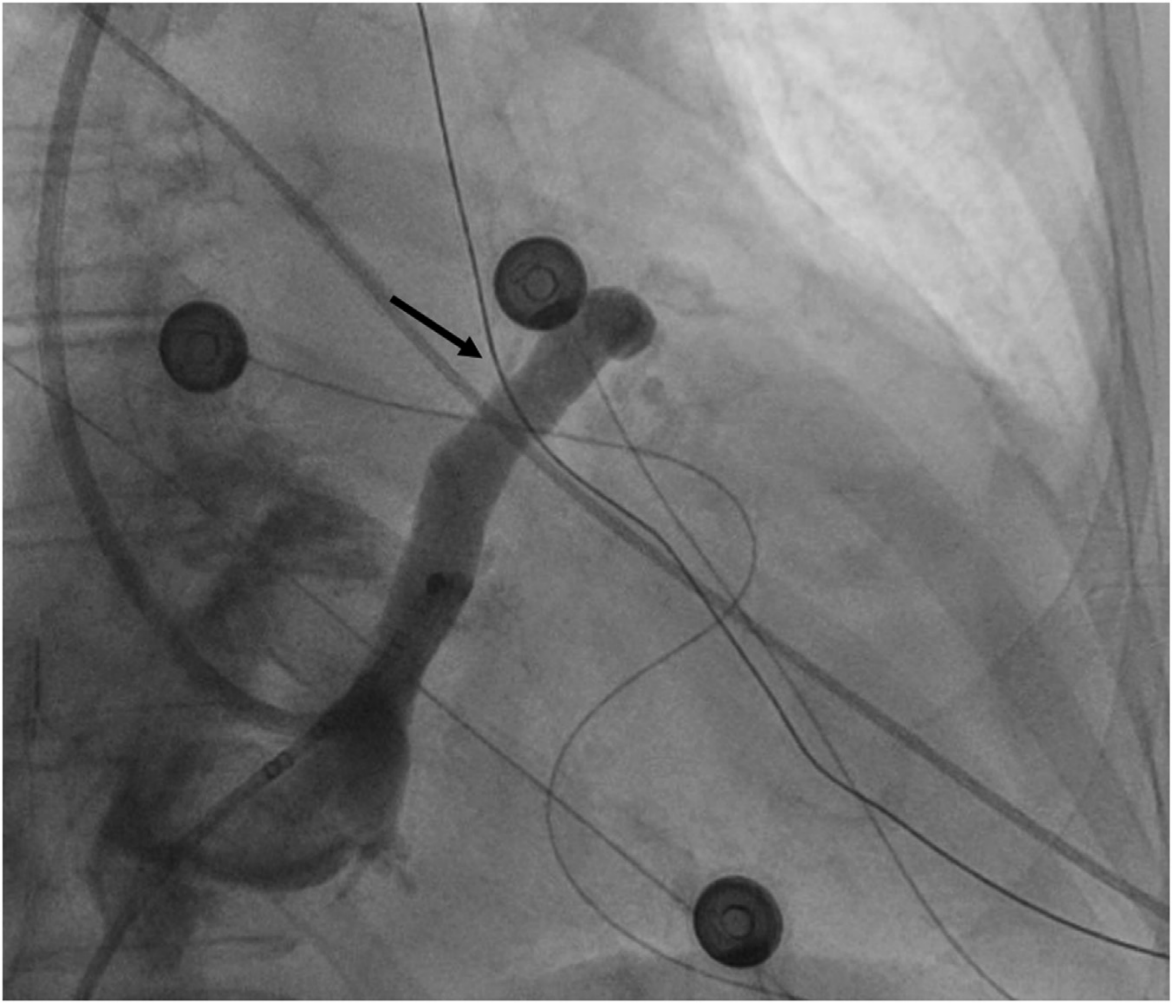
Venogram confirming the ethanol injection site within the vein of Marshall (*black arrow*).

**Figure 3:**
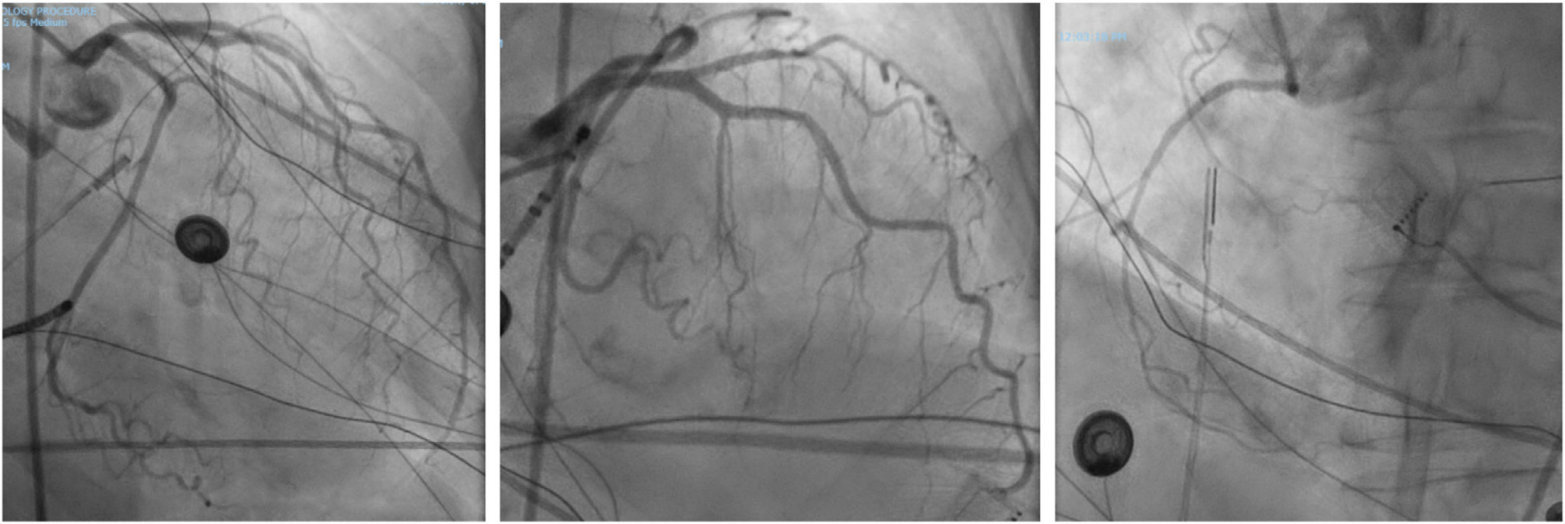
Emergent coronary angiogram showing a left-dominant system with no obvious evidence of coronary artery obstruction or spasm.

A coronary sinus venogram confirmed the ethanol injection site within the VOM (Figure 2). Emergent coronary angiography showed a left-dominant system with no obstructive coronary artery disease or spasm (Figure 3). Emergent transesophageal echocardiography revealed severe RV dysfunction, a left ventricular EF (LVEF) of 40%–45%, severe tricuspid regurgitation, and a new small pericardial effusion without evidence of tamponade. Right heart catheterization demonstrated elevated biventricular filling pressures, narrowed pulmonary artery pulse pressure, and low cardiac output.

### Management

The patient was found to be in AF with rapid ventricular response and underwent direct-current cardioversion at 360 J, restoring sinus bradycardia. Owing to her underlying shock, a left axillary temporary pacemaker was placed, set to AAI at 80 beats per minute. Given right heart catheterization findings concerning cardiogenic shock and low cardiac output, an intra-aortic balloon pump (IABP) was placed, and the patient was transferred to the cardiovascular intensive care unit (ICU) for ongoing management.

### Outcome and follow-up

**Figure 4:**
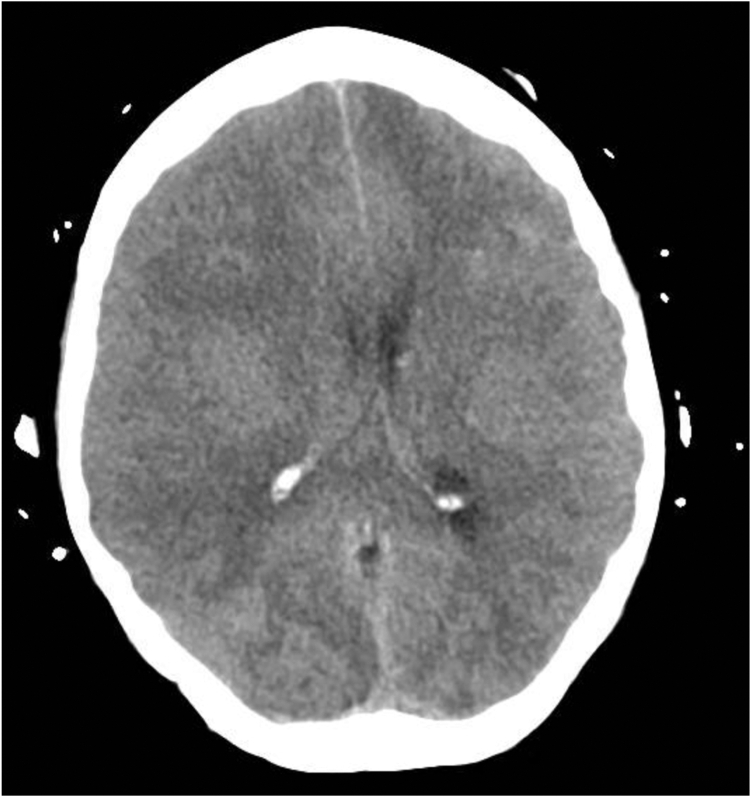
Brain computed tomography demonstrating severe diffuse anoxic brain injury, cerebral edema, midline shift, and transtentorial herniation.

On arrival at the cardiovascular ICU, the patient was on norepinephrine, dobutamine, and IABP at 1:1. Transthoracic echocardiogram demonstrated a decline in LVEF to 35%–45% compared with a previous measurement of 45%–50%. Global hypokinesis was noted. The RV was also enlarged with reduced systolic function. Initial computed tomography (CT) on arrival to the ICU showed signs concerning for hypoxic-ischemic injury in the setting of cardiogenic shock, with mild mass effect and partial effacement of the right lateral ventricle, but no herniation or acute bleeding. Follow-up CT at 24 hours revealed diffuse anoxic brain injury, cerebral edema, near-complete effacement of the right lateral ventricle, and evidence of transtentorial herniation (Figure 4). The electroencephalogram confirmed severe diffuse brain dysfunction, and neurology concluded the prognosis was extremely poor. After discussions with the family, care was withdrawn, and the patient passed away shortly thereafter. The family requested an autopsy owing to the unexplained shock after the procedure. Gross examination during autopsy did not identify a clear cause of the shock. However, there was evidence of subendocardial hemorrhage around the VOM. Despite extensive evaluation, no definitive explanation for the shock could be determined.

## Discussion

The ligament of Marshall is known to harbor both sympathetic and parasympathetic nerve fibers that contribute to the perpetuation of AF. Ethanol infusion into the VOM, a remnant of the superior vena cava and key component of the ligament of Marshall, may eliminate these AF triggers.^5^ VOM ethanol ablation during catheter-based procedures for persistent AF has been shown to be superior to standard ablation in reducing atrial tachycardia and AF burden, as well as recurrence. VOM ethanol infusion has an overall low rate of serious complications, estimated at ∼0.6%. Reported serious complications include tamponade, stroke, anaphylactic shock, AV block, and left atrial appendage isolation.^3^ To the best of our knowledge, this is the first reported case of VOM ethanol ablation leading to acute cardiogenic shock and death. In our case, the patient had diffuse ST elevations and hypotension during the VOM ethanol infusion. Differential diagnosis included air embolism, AV malformation causing alcohol entry into the arterial system, and coronary vasospasm or thrombus with spontaneous recannulation. The circumflex artery and the VOM are particularly anatomically close within the left lateral ridge of the left atrium. This proximity has clinical implications. There has been 1 previous case report describing coronary vasospasm during VOM ethanol ablation, where a patient developed ST-segment elevations and hemodynamic instability approximately 30 minutes after ethanol infusion.^4^ Interestingly, coronary angiography and coronary sinus venography during this case did not reveal any significant abnormalities or clear evidence of active coronary vasospasm, although a left-dominant coronary system was noted. It remains possible that a transient coronary artery vasospasm or brief thrombotic occlusion with spontaneous recanalization may have occurred, resulting in myocardial ischemia. This is further supported by a worsening LVEF of 35%–45% observed on repeat transthoracic echocardiogram upon arrival to the ICU.

In addition, a massive pulmonary embolism was considered, but felt unlikely given uninterrupted systemic anticoagulation and was ultimately ruled out during autopsy.

Finally, in this particular case, a notably higher dose of ethanol was administered—10 mL of 100% ethanol delivered over 3–4 minutes. This stands in contrast to previous trials, such as the VENUS trial, where significantly lower doses were used. In VENUS, up to 4 mL of ethanol was administered in divided doses depending on the anatomic features of the VOM.^2^ Although the high ethanol dose in this case raises concern for direct ethanol toxicity as a contributor to cardiogenic shock, many high-volume centers have since adopted protocols using higher doses than those in early trials without reports of increased adverse effects. This is because the anatomic characteristics of the VOM permit the use of higher ethanol volumes than other procedures, such as alcohol septal ablation, where typically only 1–3 mL of alcohol is injected into a target septal perforator artery.^6^ VOM is an epicardial vein accessible via retrograde ethanol infusion; therefore, a higher volume can be delivered in a more localized and controlled manner, potentially minimizing systemic effects.^7^ This has led to high-volume centers modifying their protocols to use higher ethanol doses than those used in early studies, tailoring the volume and rate of infusion to the patient’s VOM anatomy. For instance, 1 study reported 3 successive injections of 1–3 mL each (totaling 3–9 mL) over 1 minute, with repeat VOM angiography after each injection to confirm balloon stability.^3^ In another case, after anatomic assessment, 3 infusions of 3 mL of 98% ethanol were delivered slowly over 1 minute each.^8^ Other reports describe infusions of up to 9 mL of 95% ethanol over 5 minutes.^9^ This wide range of practice highlights the need for standardized dosing protocols and further research to ensure both efficacy and safety of VOM ethanol infusion. Nonetheless, a slower infusion rate and lower total ethanol volume are likely to be safer, and this highlights the importance of understanding patient-specific anatomy. Preprocedure CT and electroanatomic mapping can perhaps help delineate the relationship of the VOM with the coronary arteries and other collateral structures. Armed with this anatomic information, operators may be able to tailor the volume and rate of ethanol infusion to minimize risk while ensuring procedural efficacy.

Although the exact cause of our patient’s initial decompensation remains unclear, it is likely that hypotension and shock contributed to her diffuse neurologic findings and ultimately led to her death. This underscores the critical importance of operator vigilance during VOM ablations. Understanding the patient’s unique anatomy may help tailor the approach and improve safety. Continuous hemodynamic monitoring is essential throughout the procedure, and immediate access to expert interventionists for emergent angiography, along with mechanical support devices such as an IABP or extracorporeal membrane oxygenation, is crucial for managing complications and ensuring patient safety.

## Conclusion

To the best of our knowledge, this is the first reported case where VOM ethanol ablation led to acute cardiogenic shock and death. Although VOM ethanol ablation is a highly effective treatment for atrial tachyarrhythmias, operators must remain vigilant for rare, potentially fatal complications, such as cardiogenic shock and death. These serious risks underscore the importance of close monitoring of hemodynamics throughout the procedure, ensuring early detection of any adverse events. Constant observation of vital signs is crucial for prompt intervention. In addition, it is vital to have access to expert interventionists who can perform an emergent angiogram if complications arise, allowing for rapid identification and management of any vascular or structural issues. Furthermore, preparedness for the potential need for mechanical support devices, such as an IABP or extracorporeal membrane oxygenation, is essential in case of hemodynamic collapse. Awareness of the patient’s unique anatomy can help customize the approach, including determining the appropriate volume of ethanol to inject, and enhance safety. To ensure safety, it is recommended to use the lowest effective dose, administered in carefully divided increments, to minimize risks and ensure patient safety. By maintaining vigilance and ensuring readiness for advanced interventions, the risks associated with VOM ethanol ablation can be mitigated, enhancing patient safety during the procedure.

## Disclosures

The authors have no conflicts of interest to disclose.
